# Immune function in newborns with *in-utero* exposure to anti-TNFα therapy

**DOI:** 10.3389/fped.2022.935034

**Published:** 2022-08-31

**Authors:** Batia Weiss, Shomron Ben-Horin, Atar Lev, Efrat Broide, Miri Yavzori, Adi Lahat, Uri Kopylov, Orit Picard, Rami Eliakim, Yulia Ron, Irit Avni-Biron, Anat Yerushalmy-Feler, Amit Assa, Raz Somech, Ariella Bar-Gil Shitrit

**Affiliations:** ^1^Division of Pediatric Gastroenterology and Nutrition, Edmond and Lily Safra Children’s Hospital, Ramat Gan, Israel; ^2^Sackler Faculty of Medicine, Tel Aviv University, Tel Aviv, Israel; ^3^Chaim Sheba Medical Center, Institute of Gastroenterology, Ramat Gan, Israel; ^4^Pediatric Immunology Laboratory, Chaim Sheba Medical Center, Ramat Gan, Israel; ^5^Division of Pediatric Gastroenterology, Assaf-Harofe Medical Center, Ramat Gan, Israel; ^6^Souraski Medical Center, Institute of Gastroenterology, Tel Aviv, Israel; ^7^Division of Gastroenterology, Rabin Medical Center, Petah Tikva, Israel; ^8^Division of Pediatric Gastroenterology, Souraski Medical Center, Dana Duek Children’s Hospital, Tel Aviv, Israel; ^9^Schneider Children’s Hospital, Institute of Pediatric Gastroenterology, Hepatology and Nutrition, Petah Tikva, Israel; ^10^The Juliet Keidan Institute of Pediatric Gastroenterology and Nutrition, Shaare Zedek Medical Center, The Hebrew University of Jerusalem, Jerusalem, Israel; ^11^Department of Pediatrics A, Edmond and Lily Safra Children’s Hospital, Ramat Gan, Israel; ^12^Medical School, The Hebrew University, Jerusalem, Israel

**Keywords:** vaccination response, T-cell function, T-cell receptor excision circles, immunoglobulins, anti-TNFα, azathioprine

## Abstract

**Background and aim:**

Anti-TNFα is measurable in infants exposed *in utero* up to 12 months of age. Data about the exposure effect on the infant’s adaptive immunity are limited. We aimed to prospectively evaluate the distribution and function of T and B cells, in infants of females with inflammatory bowel disease, *in utero* exposed to anti-TNFα or azathioprine.

**Methods:**

A prospective multi-center study conducted 2014–2017. Anti-TNFα levels were measured in cord blood, and at 3 and 12 months. T-cell repertoire and function were analyzed at 3 and 12 months by flow-cytometry, expression of diverse T cell receptors (TCR) and T-cell receptor excision circles (TREC) quantification assay. Serum immunoglobulins and antibodies for inactivated vaccines were measured at 12 months. Baseline clinical data were retrieved, and 2-monthly telephonic interviews were performed regarding child infections and growth.

**Results:**

24 pregnant females, age 30.6 (IQR 26.5–34.5) years were recruited, 20 with anti-TNFα (infliximab 8, adalimumab 12), and 4 with azathioprine treatment. Cord blood anti-TNFα was higher than maternal blood levels [4.3 (IQR 2.3–9.2) vs. 2.5 (IQR 1.3–9.7) mcg/ml], declining at 3 and 12 months. All infants had normal number of B-cells (*n* = 17), adequate levels of immunoglobulins (*n* = 14), and protecting antibody levels to Tetanus, Diphtheria, Hemophilus influenza-B and hepatitis B (*n* = 17). All had normal CD4+, CD8+ T-cells, and TREC numbers. TCR repertoire was polyclonal in 18/20 and slightly skewed in 2/20 infants. No serious infections requiring hospitalization were recorded.

**Conclusion:**

We found that T-cell and B-cell immunity is fully mature and immune function is normal in infants exposed *in utero* to anti-TNFα, as in those exposed to azathioprine. Untreated controls and large-scale studies are needed to confirm these results.

## Introduction

Women with inflammatory bowel diseases (IBD) often require biologic therapy with anti-tumor-necrosis-factor- alpha (anti-TNFα) medications during their reproductive years to control disease activity and optimize pregnancy outcomes; however, some anti-TNFα medication cross the placenta and drug level may be measurable in the infant’s blood up to 1 year (1, 2). This fact raised major concerns about the long-term impact of *in utero* exposure to anti-TNFα on the neonatal immune system and risk of infections (3, 4). A case from 2010, reporting death of an infant exposed *in utero* to biological therapy who received at age 3 months the Bacillus-Calmette-Guerin vaccine, together with the evidence of drug persistence in the infants, have led to the current recommendation to avoid live vaccines in infants exposed *in utero* to anti-TNFα medications (5, 6). In-depth data about the effect of anti-TNFα exposure of newborns on their adaptive immunity, including humoral, and cellular components, are limited and the results are conflicting (7–13).

Azathioprine exposure during pregnancy was not associated with fetal outcomes or increased infection risk during the first year, and no serum metabolites were detected 6 weeks after delivery (14).

In addition to the assays available for evaluation of the capacity of T and B-cell immunity (15–21), a sensitive and specific surrogate marker to assess both number and function of T-cells is T-cell-receptor excision circles (TREC) quantification assay (18, 19). TRECs are formed during T-cell receptor gene rearrangement in developing T-lymphocytes (TREC) in the thymus, and therefore, are a marker for recently formed TREC. Absence or markedly reduced TREC levels indicate a low level of newly formed TREC or clonally expanded T-cells, and therefore are used as a screening test in Israel for severe type of T-cell immunodeficiency since 2015 (20, 21).

Herein, we aimed to prospectively evaluate the distribution and function of T-cells, immunoglobulin levels and the response to inactivated vaccines, from birth to 12 months of age in infants of females with IBD exposed to anti-TNFα *in utero*, compared to azathioprine exposure.

## Materials and methods

### Study design

Consecutive pregnant females attending the participating centers, with an established IBD diagnosis, treated with anti-TNFα or AZA/6MP were offered to participate in a prospective multicenter study from January 1st 2014 till December 31st 2017. Upon obtaining an informed consent, the patients provided demographic details, disease characteristics, and medication history. Enrollment could occur at any point during pregnancy, and the medical treatment was continued according to the treating gastroenterologist’s decision, irrespective of the study. Disease activity during pregnancy was defined according to the physician global assessment.

At delivery, two samples of cord blood and one sample of maternal blood for anti- TNFα serum levels were collected. Additional samples were drawn at age 3 months for anti-TNFα drug level, and T-cell repertoire and function, and at age 12 months for T-cell repertoire and function, serum immunoglobulin levels and antibody titers for inactivated vaccines: Anti- Hbs, HiB, tetanus toxoid and diphtheria. Infants exposed to AZA/6MP underwent the same tests except drug levels. Clinical data including demographics, IBD history, medications, gestational and delivery details, were retrieved from the medical charts. A telephonic infant follow-up was performed every 2 months, including growth data, history of febrile diseases, infections, hospitalizations, immunizations, medications, developmental milestones, and the presence of new medical conditions or malformations. The immunization schedule in Israel includes hepatitis B vaccines at birth, ages 1–2 and 6 months; diphtheria–tetanus–pertussis, inactivated polio vaccine, and rotavirus at 2, 4, 6 months; HiB at 2, 4, 6, 12 months and Pneumococcal vaccine at 2, 4, and 12 months.

Since no untreated IBD females could be recruited as controls, we retrospectively obtained results of the newborn screening for TREC number at delivery from 8 infants of untreated women with IBD and compared them to the TREC number at delivery of the study groups.

The study was approved by the ethical committees of the participating hospitals.

### Laboratory tests

Cord blood and maternal blood for anti-TNFα levels (Infliximab or Adalimumab) and antibodies to anti-TNFα were obtained in SST- serum separator tubes, refrigerated at 4°C and delivered in 4°C to a central laboratory at Sheba Medical Center. Infliximab and adalimumab drug and antibodies levels were measured by employing the previously described infliximab or adalimumab ELISA assay, developed at Sheba’s gastroenterology laboratory (22, 23).

Absolute numbers and percentages of lymphocytes were quantified by assessment of cell surface markers using immunofluorescent staining and flow cytometry (Epics V; Beckman Coulter, Hialeah, FL, United States) with antibodies purchased from Beckman Coulter. TREC in Guthrie cards was detected through the Israeli SCID newborn screening program that uses the commercial EnLite™ Neonatal TREC kit (Wallac Oy, Mustionkatu 6, FI-20750 Turku, Finland), as previously described (21). The following TREC analysis was performed on peripheral blood by using DNA extracted from the study patients’ PBMCs. The amount of signal joint (sj) TREC copies per DNA content was determined by real-time quantitative PCR as previously described (24). Surface expression of individual TCRVβ families was analyzed using flow cytometry and a set of TCRVβ specific fluorochrome labeled monoclonal antibodies as previously described (24). Normal control values were obtained from the IOTest Beta Mark-Quick Reference Card (Beckman Coulter).

B-cell function was determined using quantification of the immunoglobulin isotypes and specific antibody responses to immunizations.

At age 12 months, serum IgG, IgM, and IgA were measured using standard nephelometry. A sample of 3 mL Infants’ blood was used for measurement of serologic titers for tetanus toxoid (VaccZyme Human Anti Tetanus Toxoid IgG EIA kit, Binding site), Hib (VaccZyme Human Anti Haemophilus Influenza EIA kit, Binding site) and diphtheria (VaccZyme Human Anti Diphtheria Toxoid IgG EIA kit, Binding site), at the immunology laboratory, Sheba medical center, according to the manufacturer instructions. Serologic titers of anti-HbS antibodies were performed using ARCHITECT Anti-HBs (Abbott Ireland, Diagnostics Division, Sligo, Ireland) at the Gastroenterology laboratory.

The comparison of the different immunologic studies performed in the study groups was to standard laboratory normal ranges.

### Statistical analysis

Continuous variables are presented as median with interquartile range (IQR). Categorical variables are presented as *N* (%). Differences between groups were assessed using a Fisher’s exact test and Kruskal–Wallis test, for categorical and continuous data, respectively. A *P*-value of < 0.05 was considered statistically significant. Statistical analyses were performed using SAS 9.4 software (Cary, NC, United States).

## Results

Twenty-four pregnant females, 20 with anti-TNFα (infliximab 8, adalimumab 12) and 4 with azathioprine treatment, were recruited from 5 hospitals in Israel. The clinical and demographic characteristics of the patients are shown in Table 1. Additional medications to anti-TNFα included azathioprine (*n* = 3), 5-ASA (*n* = 3), and corticosteroids (*n* = 1). Therefore, we present the results of combination therapy of anti-TNFα and azathioprine as a separate group. Anti-TNFα was discontinued between weeks 24–34 in 15, and continued throughout pregnancy in 5 females (3 infliximab, 2 adalimumab), with no difference in discontinuation time between infliximab and adalimumab (median 30, IQR 30–32 vs. 29.5, IQR 25–32, *p* = 0.53). At delivery the median cord blood level and maternal blood level were 4.3 (IQR 2.3–9.2) and 2.5 (IQR 1.3–9.7) mcg/ml, respectively (*p* = 0.6)]. Maternal levels were higher in infliximab than adalimumab treated females [median 10.4 (IQR 4.3–15.4), 1.3 (IQR 0.3–2.5), *p* = 0.002)], with corresponding higher cord blood levels [median 8.8 (IQR 4.5–13.5), 1.3 (IQR 0.3–2.5) mcg/ml, *p* = 0.02]. At ages 3 (*n* = 13) and 12 months (*n* = 15), the median infants’ blood levels were 0.36 (IQR 0.1–1.34) mcg/ml and 0.06 (IQR 0.04–0.1) mcg/ml, respectively.

**TABLE 1:** Demographic and clinical characteristics of the study group.

	Total *N* = 24	Anti-TNFα *N* = 17	Azathioprine *N* = 4	Anti-TNFα + Azathioprine *N* = 3	*P*
Age at pregnancy [years, *(median, IQR*)]	32.2 (27.0–34.6)	30.1 (26.1–33.4)	33.7 (33.3–35.9)	35.0 (26.1–33.4)	0.06
**Diagnosis**					
Crohn’s disease	15	9	3	3	0.59
Ulcerative colitis	8	7	1	0	
IBD-Undetermined	1	1	0	0	
Age of diagnosis [years, *(median, IQR*)]	21.5 (18.0–26.5)	20 (18.0–24.5)	25.5 (21.5–29.0)	27.0 (15.0–27.0)	0.25
**Disease activity (pregnancy), *N* (%)**					
Remission	19 (79)	13 (76.5)	4 (100)	2 (66.7)	0.76
Mild	4 (16.7)	3 (17.6)	0	1 (33.3)	
Moderate	0	1 (5)	0	0	
**Delivery mode, *N* (%)**					
Vaginal	12 (50)	9 (52.9)	2 (50)	1 (33.3)	0.82
Cesarean	12 (50)	8 (47.1)	2 (50)	2 (66.6)	
**Delivery week *(median, IQR*)**	38.9 (38.0–39.7)	38.8 (38.0–39.5)	39.4 (38.5–40.3)	37.4 (37.4–38)	0.082

**FIGURE 1:**
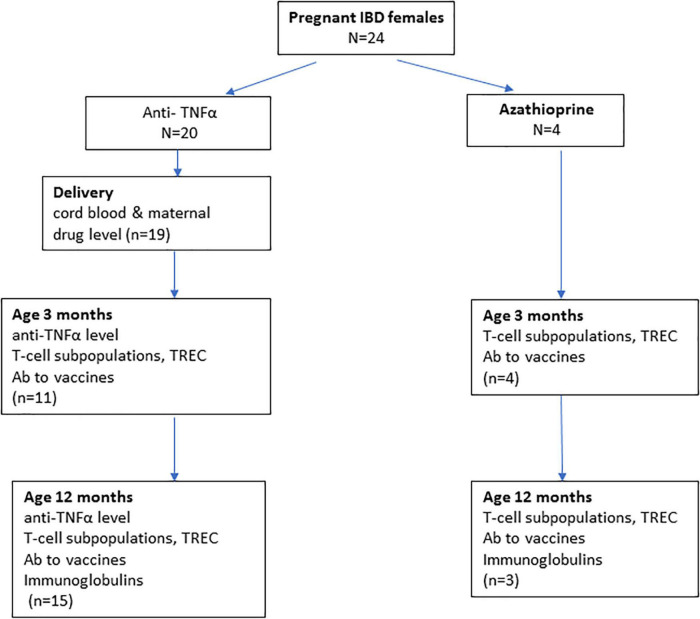
Immune function tests performed at each age of the study groups.

Not all newborns had blood samples available at both time points due to mothers’ refusal (*n* = 4 at age 3 months) and/or paucity of blood quantity withdrawn (9 infants at 3 months, 6 infants at 12 months). Only 4 mothers refused infants’ blood testing at age 3 months, but agreed at age 12 months. All had evaluation of the T-cell function at least once (Figure 1).

### Pregnancy outcome and newborn follow-up

Two infants exposed to anti-TNFα were born preterm (gestational weeks 30,36, birth weights 1,300, 2,480 gr). Two minor congenital malformation (1 pelvic kidney, 1 umbilical cord containing 2 blood vessels) were diagnosed in infants exposed to azathioprine (Table 2).

**TABLE 2:** Pregnancy outcome and 12-month follow-up of infants exposed *in utero* to anti-TNFα, azathioprine or combination therapy.

*N* (%)	Total *N* = 24	Anti-TNFα *N* = 17	Azathioprine *N* = 4	Anti-TNFα + Azathioprine *N* = 3	*P*
Male gender	14 (58)	8 (47.1)	3 (75)	3 (100)	0.17
Birth weight [Kg *(median, IQR*)]	3.0 (2.6–3.4)	3.1 (2.7–3.4)	3.1 (2.7–3.7)	2.0 (2.0–3.1)	0.22
Congenital malformations*	1 (4.2)	1 (5.9)	0	0	0.80
Post-natal antibiotics	6 (25)	3 (17.6)	1 (25)	2 (66.7)	0.20
Phototherapy	2 (8.7)	2 (10)	0	0	0.62
**Breast feeding- 3 mon**					
Full	9 (38)	7 (41.2)	2 (50)	0	0.35
Partial	4 (16.7)	3 (17.6)	1 (25)	0	
Weight- 12 mon [Kg *(median, IQR*)]	9.8 (8.7–10.2)	9.7 (8.9–10.1)	9.3 (8.3–10.3)	10.2 (9.5–10.2)	0.67
Height – 12 mon [Cm *(median, IQR*)]	75.0 (73.0–77.6)	75.0 (72.0–77.0)	76.3 (74.0–77.8)	76.5 (73.6–79.0)	0.53
**Immunizations-12 mon**					
**DTP + HIB*^a^***					
None	1 (4.2)	0	1 (25)	0	0.38
2 doses	2 (8.3)	2 (11.8)	0	0	
3 doses	20 (83.3)	14 (82.4)	3 (75)	3 (100)	
4 doses	1 (4.2)	1 (5.9)	0	0	
**Hepatitis B**					
None	1 (4.2)	0	1 (25)	0	0.23
2 doses	1 (4.2)	1 (5.9)	0	0	
3 doses	22 (91.7)	16 (94.1)	3 (75)	3 (100)	
***Rota* virus**					
None	9 (37.5)	8 (47.1)	1 (25)	0	0.46
1 dose	2 (8.3)	2 (11.8)	0	0	
2 doses	1 (4.2)	1 (5.9)	0	0	
3 doses	12 (50)	6 (35.3)	3 (75)	3 (100)	

*Left pelvic kidney.

DTP, diphtheria, tetanus, pertussis; HIB, hemophilus influenza.

Despite a recommendation to avoid live-vaccines with exposure to anti-TNFα, 9/20 (45%) infants received a full 3-dose *Rota virus* vaccination and 3 (15%) infants 1–2 vaccine doses by age 6 months, with no reported complications. One mother in the azathioprine group refused all immunizations.

There were no hospitalizations due to infections during the first year of life. Two infants exposed to anti-TNFα monotherapy (1 discontinued at week 30, 1 continued throughout pregnancy), had one event of pneumonia, and one was clinically diagnosed with pertussis despite immunizations, at age 10 months. Infants exposed to combination therapy did not have significant infections (Table 3). There was no correlation between the appearance of any infection and cord blood anti-TNFα level.

**TABLE 3:** Acquired infections of the newborns during 12 months follow-up.

Infection	Total no. episodes	0–6 months	6–12 months
URTI* (± fever)	8	3	5
Otitis media	7	1	6
Conjunctivitis	4	–	4
Aphtous stomatitis	3	–	3
Pneumonia	2**	–	2
Fever/Rash	7	2	5
Pertussis	1**	–	1
Acute Gastroenteritis	1	3	4

*URTI, upper respiratory tract infection.

**Maternal treatment: pneumonia 1- anti-TNFα, discontinued week 30; pneumonia 2- anti-TNFα, continued throughout pregnancy; pertussis- anti TNFα, continued throughout pregnancy.

### Immune function

Infants born since October 2015 underwent newborn screening for SCID using quantification of TREC copies from Guthrie cards, with TREC numbers above the cutoff level (> 25 TREC copies per 1.5 mm punch). Six newborns (5 with anti- TNFα monotherapy and one with azathioprine) had newborn screening at birth. The TREC number at birth was similar to newborns of females with IBD with no medication exposure during pregnancy (Table 4).

**TABLE 4:** TREC number at delivery of infants exposed *in utero* to anti-TNF alpha or azathioprine compared to unexposed infants of mother with IBD.

	Anti-TNFα *N* = 17	Azathioprine *N* = 4	No treatment *N* = 14	*P*
Male gender (%)	8 (47)	3 (75)	8 (57.1)	0.76
Birth week [Kg *(median, IQR*)]	38.8 (38.0–39.5)	39.4 (38.5–40.3)	39 (39.0–40.0)	0.36
TREC at delivery* [*(median, IQR*)]	112 (104–199) *n* = 5	197 *n* = 1	141 (53–231) *n* = 8	0.90

*Screening from October 2015.

All infants had normal B-cell numbers [3 months; median 3,913 (IQR 2,751–5,624), 12 months; 4,852 (IQR 4,014–8,716)], adequate immunoglobulin levels and normal protecting levels of antibodies to previous vaccinations (Table 5). The infant who developed pertussis despite vaccination had antibodies to other vaccination excluding possible dysfunctional B cell immunity. There was no correlation between umbilical cord anti-TNFα level and immunoglobulin levels or response to vaccination at ages 3 and 12 months.

**TABLE 5:** B-cell and T-cell function of the newborns exposed to anti-TNFα, Azathioprine and combination therapy.

	Anti-TNFα *N* = 17		Azathioprine *N* = 4		Anti-TNFα + Azathioprine *N* = 3		*P*
	Age 3 months *N* = 8	Age 12 months *N* = 13	Age 3 months *N* = 4	Age 12 months *N* = 3	Age 3 months *N* = 3	Age 12 months *N* = 2	
WBC mm^3^ Median, (IQR)	11,475 (8,800–13,910)	12,480 (10,390–14,690)	6,565 (9,100–12,230)	10,665 (9,340–14,690)	10,110 (8,040–13,080)	7,990 (6,850–9,130)	0.78^∧^, 0.15^∧∧^
**T-cells subpopulations** **Median, (IQR)**							
Lymphoctyes mm^3^	6,701 (6,020–8,705)	6,558 (5,260–8,295)	5,327 (4,651–7,429)	4,863 (3,977–5,748)	6,703 (5,258–7,456)	3,733 (2,836–4,629)	0.47^∧^, 0.06^∧∧^
CD3/mm^3^	4,862 (4,177–5,301)	4,067 (3,314–5,889)	3,746 (3,191–4,886)	3,452 (2,823–4,081)	3,419 (2,997–4,473)	2,432 (1,900–2,963)	0.20, 0.09
CD4/mm^3^	3,770 (3,217–3,195)	2,530 (2,209–3,155)	2,772 (2,512–3,585)	1,684 (1,380–1,988)	2,156 (1,193–2,480)	1,256 (1,078–1,435)	0.06, 0.04
CD8/mm^3^	1,135 (971–1,532)	1,274 (915–2,427)	1,023 (706–1,255)	1,583 (636–2,529)	938 (631–3,131)	1,063 (737–1,389)	0.51, 0.73
CD20/mm^3^	1,433 (883–2,437)	1,472 (1,399–1,746)	1,094 (1,044–1,949)	876 (716–1,035)	1,542(1,052–2,311)	774 (482–1,065)	0.96, 0.04
CD56 (neutral, NK)	6.5 (4.0–8.5)	7.00 (400–10.00)	4.00 (3.5–4.5)	5.00 (5.00–5.00)	1.0 (1.0–5.0)	3.0 (3.0–3.0)	0.11, 0.25
TCR v-beta quantific *	Normal/polyclonal	Normal/polyclonal-13, Clonal- 2	Normal/polyclonal	Normal/polyclonal	Normal/polyclonal	Normal/polyclonal	
TREC	7,280 (4,250–9,112)	6,301 (2,842–7,654)	7,998 (5,186–16,689)	6,051 (4,323–7,538)	9,974 (7,951–12,270)	5,788 (2,842–8,734)	0.24, 0.86
Immunizations **							
Anti-HiB Ab*** mg/l	0.5 (0.1–0.9)	0.9 (0.2–5.2)	0.2 (0.2–0.2)	0.5 (0.2–1.6)	0.3 (0.2–0.3)	0.6 (0.4–0.7)	0.63, 0.86
Anti-Diphtheria Ab IU/ml	0.3 (0.0–1.8)	1.1 (0.6–2.3)	0.0 (0.0–0.0)	2.4 (0.1–4.7)	0.9 (0.3–1.5)	3.2 (0.7–5.7)	0.25, 0.78
Anti-Tetanus Ab IU/ml	0.1 (0.1–0.8)	0.8 (0.3–1.4)	0.1 (0.1–0.1)	0.9 (0.3–1.5)	0.3 (0.3–0.4)	3.1 (1.6–4.6)	0.64, 0.28
HbsAb mIU/ml	NA	193.9 (29.0–1001.0)	NA	50.2 (2.1–1001.0)	NA	70.4 (68.7–72.0)	0.93^∧∧^
**Immunoglobulins mg/dl** **Median, (IQR)**							
IgG	NA	914 (812–1180)	NA	699 (455–841)	NA	787 (718–856)	0.15^∧∧^
IgA	NA	50 (44–61)	NA	36 (29–67)	NA	52 (43–62)	0.69^∧∧^
IgM	NA	75 (70–99)	NA	99 (66–113)	NA	94 (72–115)	0.75^∧∧^

*At age 3 months- available for 8 infants: 4- Anti-TNFα, 2– Anti-TNFα + azathioprine, 2- azathioprine.

**At age 3 months- available for 10 infants: 7- Anti-TNFα, 2- Anti-TNFα + azathioprine, 1-azathioprine.

***Hemophilus influenza B vaccine.

^∧^p for age 3 months.

^∧∧^p for age 12 month.

**FIGURE 2:**
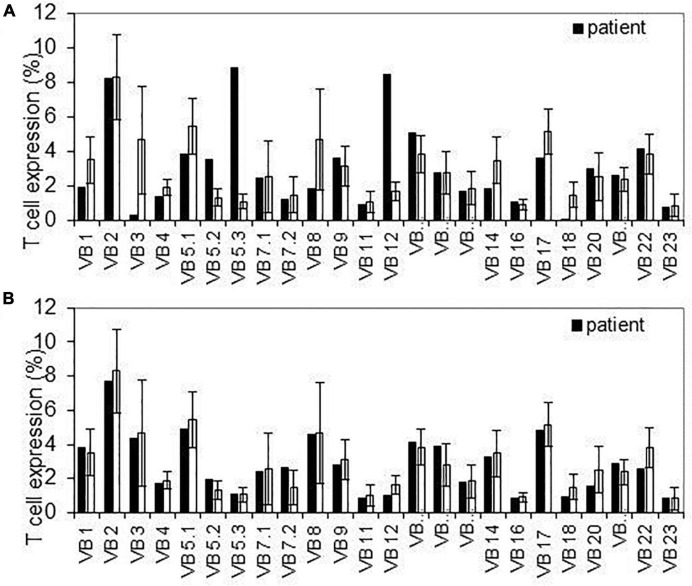
T-cell receptor repertoire detected variable segment usage for T cell receptor β. Flow cytometer analysis of surface membrane expression of 24 T cell receptor β variable segments in infants with slightly abnormal pattern (upper panel-**A**) and normal pattern (lower panel-**B**) compared to average expression on healthy controls. Error bars indicate *SD* across controls.

All infants had normal levels of CD4+, CD8 + T-cells, and TREC during the first year of life (Table 5). In order to better assess the function of the T cell adaptive immunity, TCR repertoire analysis was performed. All, beside two infants exposed to anti-TNFα, had normal representation of polyclonal repertoire (Figure 2). Two infants had a slightly skewed repertoire, with no correlation to clinical outcomes, and similar anti-TNFα cord blood and serum levels to the other infants. We did not find significant longitudinal differences during the first year of life in each infant, suggestive a full mature T-cell immunity since infancy with exposure to anti-TNFα, azathioprine or combination therapy. At age 12 months, a numerical decrease in CD4 and CD20 cells was observed in infants exposed to combination therapy compared to monotherapy (Table 5). However, all differences were within the normal range cell number, with no difference between exposure to infliximab or adalimumab (Table 6). Anti-TNFα cord blood levels correlated negatively with CD4 T-helper cell number (*r* = −0.85, *p* = 0.003, *n* = 9) and positively with the number of CD8 T-cells (*r* = 0.72, *p* = 0.02, *n* = 10) at age 3 months but not 12 months (*r* = −0.38, *p* = 0.27, *n* = 10, and *r* = −0.28, *p* = 0.43, *n* = 10, respectively). Those differences, although statistically significant, were all within the normal range of the T-cell subsets number.

**TABLE 6:** B-cell and T-cell function of the newborns exposed to Infliximab, Adalimumab, Azathioprine, or combination therapy.

	Infliximab *N* = 7		Adalimumab *N* = 10		Azathioprine *N* = 4		Anti-TNFα + Azathioprine *N* = 3		*P*
	3 mon *N* = 4	12 mon *N* = 7	3 mon *N* = 4	12 mon *N* = 9	3 mon *N* = 4	12 mon *N* = 3	3 mon *N* = 3	12 mon *N* = 2	
WBC mm^3^ Median, (IQR)	11,990 (7,880–12,800)	14,690 (13,440–16,520)	10,960 (9,170–15,020)	10,605 (10,140–12,865)	9,595 (8,600–12,095)	10,665 (9,340–14,690)	10,110 (8,040–13,080)	10,110 (6,850–9,130)	0.81^∧^ 0.19^∧∧^
**T-cells subpopulations Median, (IQR)**									
Lymphoctyes mm^3^	5,327 (4,651–7,429)	8,295 (5,974–9,529)	5,327 (4,651–7,429)	6,559 (5,460–7,144)	5,327 (4,651–7,429)	4,863 (3,977–5,748)	5,327 (4,651–7,429)	3,733 (2,836–4,629)	0.31^∧^0.13^∧∧^
CD3/mm^3^	4,837 (3,298–5,618)	5,889 (4,242–6,519)	4,887 (4,309–4,983)	4,001 (3,659–4,855)	3,746 (3,191–4,886)	3,452 (2,823–4,081)	3,418 (2,997–4,473)	2,432 (1,900–2,963)	012^∧^0.15^∧∧^
CD4/mm^3^	3,766 (2,864–4,668)	2,530 (2,151–3,145)	3,770 (3,217–3,915)	2,498 (2,289–3,324)	2,772 (2,512–3,585)	1,684 (1,380–1,988)	2,156 (1,193–2,480)	1,265 (1,078–1,435)	0.63^∧^0.09^∧∧^
CD8/mm^3^	1,334 (1,039–1,726)	2,151 (1,659–2,919)	1,091 (971–1,246)	1,189 (1,003–1,851)	1,023 (706–1,255)	1,583 (636–2,529)	938 (631–3,131)	1,063 (737–1,389)	0.77^∧^0.61^∧∧^
CD20/mm^3^	990 (776–2,383)	1,239 (836–1,410)	1,541 (1,326–2,491)	1,530 (1,446–1,791)	1,094 (1,044–1,949)	876 (716–1,035)	1,542 (1,052–2,310)	774 (482–1,065)	0.77^∧^0.04^∧∧^
CD56 (neutral, NK)	3.00 (2.0–5.0)	7.0 (4.0–10.0)	8.0 (8.0–9.0)	7.0 (4.4–10)	4.0 (3.5–4.5)	5.0 (5.00–5.00)	1.0 (1.0–1.5)	3.0 (3.0–3.0)	0.03^∧^0.43**^∧∧^**
TCR v-beta quantific *	Normal/polyclonal	Normal/polyclonal	Normal/polyclonal	Normal/polyclonal	Normal/polyclonal	Normal/polyclonal	Normal/polyclonal	Normal/polyclonal	
TREC	7,280 (6,332–7,814)	6,565 (6,301–6,978)	5,798 (3,523–9,112)	3,310 (1,950–7,225)	7,998 (5,186–16,689)	6,051 (4,323–7,538)	9,975 (7,951–12,270)	5,788 (2,842–8,734)	0.39^∧^0.55^∧∧^
Immunizations **									
Anti–HiB Ab*** mg/l	0.2 (0.2–0.2)	5.2 (2.5–10.6)	0.6 (0.1–0.9)	0.4(0.2–3.0)	0.2 (0.2–0.2)	0.5 (0.2–1.6)	0.3 (0.2–0.3)	0.6 (0.4–0.7)	0.82^∧^0.72^∧∧^
Anti-Diphtheria Ab IU/ml	0.1 (0.0–0.2)	1.7 (1.5–4.8)	0.6 (0.3–1.8)	0.7 (0.3–1.1)	0.0 (0.0–0.0)	2.4 (0.1–4.7)	0.9 (0.3–1.5)	3.2 (0.7–5.7)	0.17^∧^0.43^∧∧^
Anti-Tetanus Ab IU/ml	0.2 (0.1–0.4)	1.4 (0.3–2.6)	0.1 (0.1–0.8)	0.6 (0.4–0.9)	0.1 (0.1–0.1)	0.9 (0.3–1.5)	0.3 (0.3–0.4)	3.1 (1.6–4.6)	0.74^∧^0.35^∧∧^
HbsAb mIU/ml	NA	29.0 (23.3–43.4)	NA	404.5 (43.5–1001.0)	NA	50.2 (2.1–1001.0)	NA	70.7 (68.7–72.0)	0.27^∧∧^
**Immunoglobulins mg/dl, Median, (IQR)**									
IgG	NA	914 (812–1,290)	NA	942 (704–1,180)	NA	699 (455–841)	NA	787 (718–856)	0.26^∧∧^
IgA	NA	50 (38–79)	NA	53 (44–61)	NA	36 (29–67)	NA	52 (43–62)	0.84^∧∧^
IgM	NA	75 (57–99)	NA	75 (70–111)	NA	99 (67–113)	NA	93 (72–115)	0.81^∧∧^

*At age 3 months- available for 8 infants 4- Anti-TNFα, 2– Anti-TNFα + azathioprine, 2- azathioprine.

**At age 3 months- available for 10 infants: 7- Anti-TNFα, 2- Anti-TNFα + azathioprine, 1-azathioprine.

***Hemophilus influenza B vaccine.

^∧^p for age 3 months.

^∧∧^p for age 12 month.

## Discussion

We report a prospective and detailed immune function evaluation of infants of IBD patients, with prenatal exposure to anti-TNFα medications or azathioprine. We show, for the first time by a marker for recently formed TREC, that T-cell function is normal since birth and during follow-up. Although the main use of TREC is for exclusion of SCID and primary immune deficiencies, the normal number during the first year of life suggests, together with the measurement of T-cell subpopulations, a normal T-cell function. In addition, their B-cell number and functions are normal, with normal immunoglobulins levels and intact response to childhood vaccinations.

Children of mothers with IBD do not have an increased long-term risk for chronic or malignant diseases except IBD (25). While multiple studies reported safety of anti-TNFα medications regarding pregnancy outcomes (26–30), their influence on the immune system of the exposed infants is not completely resolved. The mechanism of action of anti-TNF antibodies is still a matter of debate, and differs between peripheral and mucosal cells, as well as *in vivo* and *in vitro*. Their effects are mediated through complex interactions with various immune cell populations involved in the pathogenesis of IBD (31, 32), and therefore, there is a rationale for testing the immune function of *in utero* exposed infants.

Bortlik et al. (10), followed 25 exposed children to age 34 months and reported normal antibody responses against tetanus, S. pneumonia, diphtheria, rubella, morbilli, and paritits (*n* = 15), but low antibodies to HiB in 6/15, mild reduction in immunoglobulin levels in 7/15 (41%), and normal lymphocyte subpopulation counts. However, the immunological tests were not performed within constant times from vaccination or from birth. Despite the recommendations, two thirds of the children received BCG vaccine but only 3 developed larger skin reaction and 1 had lymphadenopathy. Duricova et al. (12), reported an adequate antibody response for tetanus, diphtheria, streptococcus pneumonia, measles, and rubella in over 95% of infants, for HiB in 65% and for Mumps in 75% of 49 children exposed *in utero* to anti-TNFα compared to healthy controls, median ages 35 and 50 months. Fifteen infants received BCG vaccination within the first week of life, of them 4 developed large skin reactions and one axillar lymphadenopathy, compared to 2 of the controls. One report from the PIANO registry including 12 *in utero* exposed infants showed normal IgA and IgG levels, low IgM levels in 5/10 (50%), but a normal antibody response to tetanus and Hib vaccines at age 6–28 months in 92%, and no increase in infection rates (7). Another study from the same registry, found no difference in adequate antibody titers to Hib and tetanus between infants exposed or unexposed to anti-TNFα *in utero*; 71% of exposed (*n* = 41) and 50% of unexposed (*n* = 8), 80% (*n* = 8) and 75% (*n* = 8) of exposed and unexposed children, respectively (11). Importantly, 35 exposed infants received the *Rotavirus* vaccines, with reaction of fever in 6 and diarrhea in one, comparable to the general population. A recent French study (33) reported that 13% of infants exposed to anti-TNFα medications (43% throughout pregnancy) were vaccinated against tuberculosis before age 6 months, with no case of disseminated BCG infection, and 12 were vaccinated against Measles–Mumps-Rubella before age 9 months, with no events of severe infection. This study provides no data on the serum anti-TNFα levels in the exposed infants. A prospective small study of 7 exposed infants reported a normal number but less mature B and helper T-phenotype that normalized within 12 months, decreased T-reg cells at birth, and normal immunoglobulins, vaccine responses, and T-cell proliferation to mitogens. However, a decreased response after mycobacterial challenge at birth was noted, with no complete recovery after drug removal, suggesting that the immune system activation upon mycobacterial challenge may be compromised (9). Kattah et al. (8), used multiparameter flow cytometry on 22 infants with different *in utero* exposures from the PIANO registry at age 12 months; (1 = no exposure, 4 certolizumab, 11 infliximab/adalimumab, 4 infliximab/adalimumab/immunomodulator, 4 certolizumab/immunomodulator). In those small different exposure groups, B and T lymphocyte subsets were preserved in all, with no increased risk of infections.

There were no infections requiring hospitalizations in our group of infants, even in those exposed in the third trimester. Although we do not have a control group of infection rate in the first year of life, the number of 33 infections in 24 infants, without a need for hospitalization, is within normal limits (34). In a study from the Czech- Rebulic no increase in infection rate was found in children exposed to anti-TNFα, compared to unexposed infants of non-IBD mothers (12). A recent large population- based study reported a slightly increased rate of infections in anti-TNFα exposed infants, defined by hospital admissions for infection in the first year of life, as well as increased rates for antibiotic prescriptions in the second year of life. The incidence rate ratios for infections were similarly increased when treatment was stopped before or continued during the third trimester, and was not higher with combination therapy, compared to non-biological treatment exposure and the general population (35). In another large study from Denmark *in utero* exposure to anti-TNFα but not to thiopurines resulted in an increased risk of respiratory and uro-gynecological infections during the first year (36). Both studies included infants of females with different inflammatory disorders, who may be incomparable to IBD patients. A recent study of neonatal outcomes after fetal exposure to biologics, thiopurines, combination therapy or no treatment (IBD, 1,490 pregnancies) showed no difference in infection rate during the first year of life (30).

The advantages of our study are the prospective measurements of TREC, antibody responses to vaccines, immunoglobulin levels and serum drug levels, at exact intervals since birth, and a control group of immunomodulatory treatment alone. In contrast to previous studies, we show a completely normal antibody response and normal immunoglobulin levels in all infants. BCG is not part of the vaccination program in Israel, and the only first- year live vaccine is *Rota virus*. The TREC measurement is a novel method used in our study, allowing evidence of the intact T-cell immunity since early infancy. This reinforces the indirect evidence from previous studies in which live vaccines where administered despite recommendations, that live vaccines may be considered during the first year of life in infants exposed to anti-TNFα medications *in utero*. Indeed, in our study 12 (60%) infants received at least one dose of *Rota virus* vaccine with no complications. The debate about live vaccines in those infants should consider the risk of specific infections like tuberculosis, measles mumps rubella, and *Rota virus* in different geographic areas, and the risk-benefit ratio between disease acquirement and possible vaccine complications. Measurement of drug levels prior to vaccination can assess decision making (37). Larger studies and *in vitro* challenge investigations are needed in prior to changing the recommendations.

Our study has a few limitations. Firstly, our groups of patients and controls are still small and not all tests were performed at all points for every patient, a possible cause of bias. Previous studies included similar small control groups, due to recruitment difficulties in studies involving infant blood drawing. Secondly, we were not able to recruit untreated IBD females, due to maternal reluctance to perform blood tests to the infants. However, we were retrospectively able to compare the TREC number at birth of unexposed infants of IBD females to a small number of patients form the study, showing no difference in this aspect. Thirdly, we have not examined IL-12/IFN-γ levels, relevant to mycobacterial infection.

## Conclusion

In conclusion, aspects of the adaptive immune system, including T-cell and B-cell functions were normal in infants exposed *in utero* to anti-TNFα, suggesting that a significant secondary immunodeficiency is unlikely. Further studies are required, examining response to mycobacterial challenge and the IL-12/IFN-γ pathway. We believe the live vaccine immunization schedule of anti-TNFα exposed infants, should be considered according to the geographic disease epidemiology, and the risk-benefit stratification.

## Data availability statement

The original contributions presented in this study are included in the article/supplementary material, further inquiries can be directed to the corresponding author.

## Ethics statement

The studies involving human participants were reviewed and approved by the Ethics Committee Sheba Medical Center. Written informed consent to participate in this study was provided by the participants’ legal guardian/next of kin.

## Author contributions

BW contributed to study concept and design, statistical analysis and interpretation of data, drafting of manuscript, and has approved the final draft submitted. SB-H contributed to study concept and design and critical revision of the manuscript. ALe performed the immunological tests. EB, ALa, UK, RE, YR, IA-B, AY-F, and AA contributed to patient recruitment, acquisition of data, and critical revision of the manuscript. MY and OP contributed to performance of immunological tests and critical revision of the manuscript. RS contributed to study concept and design and drafting of manuscript. AB-G contributed to study concept and design, patient recruitment, acquisition of data, and critical revision of the manuscript. All authors contributed to the article and approved the submitted version and approved the final draft submitted.
